# Albumin from *Erythrina edulis* (Pajuro) as a Promising Source of Multifunctional Peptides

**DOI:** 10.3390/antiox10111722

**Published:** 2021-10-28

**Authors:** Cleni Palma-Albino, Arturo Intiquilla, Karim Jiménez-Aliaga, Nathaly Rodríguez-Arana, Estela Solano, Eduardo Flores, Amparo Iris Zavaleta, Víctor Izaguirre, Blanca Hernández-Ledesma

**Affiliations:** 1 Grupo de Investigación BIOMIAS, Departament of Biochemistry, Facultad de Farmacia y Bioquímica, Universidad Nacional Mayor de San Marcos, Jr. Puno 1002, Lima 4559, Peru; cleni.palma@unmsm.edu.pe (C.P.-A.); aintiquillaq@unmsm.edu.pe (A.I.); nathaly.rodriguez@unmsm.edu.pe (N.R.-A.); stelasolano26@gmail.com (E.S.); efloresj@unmsm.edu.pe (E.F.); azavaletap@unmsm.edu.pe (A.I.Z.); vizaguirrep@unmsm.edu.pe (V.I.); 2 Departamento de Ciencia de los Alimentos y Tecnología Química, Facultad de Ciencias Químicas y Farmacéuticas, Universidad de Chile, Santos Dumont 946, Santiago de Chile 8380492, Chile; 3 Department of Bioactivity and Food Analysis, Instituto de Investigación en Ciencias de la Alimentación (CIAL, CSIC-UAM, CEI UAM+CSIC), Nicolás Cabrera 9, 28049 Madrid, Spain

**Keywords:** *Erythrina edulis*, legume proteins, sequential enzymatic digestion, multifunctional peptides

## Abstract

Multifunctional peptides, capable of acting on different body systems through multiple mechanisms of action, offer many advantages over monofunctional peptides, including lower adverse side effects and costs. *Erythrina edulis* (pajuro) is a legume with a large number of high-quality proteins, of which their potential as a source of antioxidant peptides has been recently reported. In this study, the behavior of these proteins under a sequential enzymatic hydrolysis with digestive and microbial enzymes was investigated by evaluating the multi-functionality of the hydrolyzates. The albumin hydrolyzates obtained after the action of pepsin, pancreatin, and Alcalase showed antioxidant, angiotensin-converting enzyme (ACE), α-amylase, α-glucosidase, and dipeptidyl peptidase (DPP)-IV inhibitory activities. The radical scavenging properties of the hydrolyzate could be responsible for the potent protective effects observed in FeSO_4_-induced neuroblastoma cells. The findings support the role of pajuro protein as an ingredient of functional foods or nutraceuticals for health promotion and the prevention of oxidative stress, hypertension, and metabolic alteration-associated chronic diseases.

## 1. Introduction

Proteins are vital macronutrients involved in body maintenance and growth; thus, the search for high-quality proteins and their inclusion into the daily diet has become a prevalent research area. To meet consumer trends in limiting the intake of animal-derived proteins, nutritionists, researchers, and food industries are exploring novel and sustainable protein sources. Among them, traditional and under-utilized plants are becoming popular as a source of proteins that provide important nutritional, technological, and functional properties [1]. In addition to these attributes, proteins also provide multiple health benefits through their impact on specific biochemical pathways. Most of these activities result from peptides encrypted within the parent protein sequence that, once released by enzymatic hydrolysis, gastrointestinal digestion, or food processing, are absorbed by intestinal cells and transported to their target organs, and then, can exert their biological effects [2]. Over the years, numerous bioactive peptides have been identified from plant foods protein hydrolyzates. Their diversified structures explain the wide range of activities demonstrated for these peptides, such as antioxidant, anti-inflammatory, anti-hypertensive, anti-microbial, anti-diabetic, and chemo-preventive activities, among others [3,4,5]. These reported effects demonstrate the potential of bioactive peptides to be used as ingredients of functional foods and/or nutraceuticals aimed at promoting health and reducing the risk of suffering from non-communicable diseases (NCDs) [6].

NCDs, such as cardiovascular and neurodegenerative disorders, diabetes, and cancer are the main cause of mortality and incapacity worldwide [7]. The environmental factors and, mainly, the diet are the major contributing factors to most of these diseases. While the intake of processed foods and sugar-sweetened beverages has been correlated with a higher risk of NCDs, a healthy diet including functional foods has been found to reduce or even prevent several of these disorders [7,8]. It has been demonstrated that most of NCDs show common etiological characteristics including oxidative stress, hypertension, inflammation, and metabolic alterations [9]. Thus, acting against one or more of these pathophysiological conditions with natural derived compounds has become a promising alternative for the prevention/management of NCDs. Among these compounds, multifunctional peptides, defined as peptides with the ability to exert more than one physiological effect by affecting several targets, represent an emerging area with multiple applications [10]. They may be considered enhancements compared with mono-functional peptides, which exert one single activity, owing to reduced negative side effects and costs [11].

*Erythrina edulis* (pajuro or chachafruto) is a legume with a wide variety of uses, from the human (seeds) and animal (forage) diet to the recovery of nitrogen from the soil. This plant is recognized by its high nutritional value, containing a superior content of high-quality and digestibility proteins (18–25%) [12]. Moreover, the seed has been found to be rich in carbohydrates (51%) and micronutrients, such as minerals (phosphorus, iron, sulfur, sodium, potassium, manganese, and calcium) and vitamins (vitamin C, thiamin, niacin, and riboflavin) [12]. In addition of its nutritional attributes, pajuro has been traditionally used for medical purposes as a diuretic, hypotonic, and osteoporosis prophylactic [13]. These attributes have been associated with the presence of phytochemicals such as saponins, alkaloids, flavonoids (mainly quercetin), and polyphenols (mainly phloroglucinol) [14]. Despite its high protein content, information concerning the biological properties of peptides contained in their sequences is still scarce and limited to antioxidant activity. Thus, in a previous study carried out in our group, the radical scavenging activity of *E. edulis* protein hydrolyzates with different enzymes was demonstrated [13], and peptides contained in the Alcalase hydrolyzate were identified as potentially responsible for the observed effects [15]. However, no data on other activities have been reported. Thus, the aim of this work was to separate pajuro protein into its different fractions and to hydrolyze them by a sequential hydrolytic reaction with both digestive and microbial enzymes. The multi-functionality of hydrolyzates was investigated, focusing on their in vitro anti-hypertensive, anti-diabetic, and neuroprotective effects on both biochemical and cell models. 

## 2. Materials and Methods

### 2.1. Materials

*E. edulis* (pajuro) seeds were collected in Otuzco–La Libertad (Peru) in 2016. Pepsin from porcine gastric mucosa (EC 3.4.23.1; 0.7 International Pharmaceutical Federation units (FIP) U/mg protein), pancreatin from porcine pancreas (0.35 FIP-U/mg protein), iron II sulfate heptahydrate, ortho-phthalaldehyde (OPA), 3,5-dinitrosalicylic acid, sodium potassium tartrate, and thiobarbituric acid (TBA) were purchased from Merck Millipore Corp. (Darmstadt, Germany). Alcalase 2.4 U/g protein from *Bacillus licheniformis* was acquired from Novozymes (Bagsvaerd, Denmark). Bovine seroalbumin (BSA), 2,2′-azino-bis(3-ethylbenzothiazoline-6-sulfonic acid) (ABTS^•+^), 6-hydroxy-2,5,7,8-tetramethylchroman-2-carboxylic acid (Trolox), disodium fluorescein (FL), 2,2′-azobis (2-amidinopropane dihydrochloride) (AAPH), angiotensin converting enzyme (ACE) from lung rabbit, hippuryl-histidyl-leucine (HHL), captopril, type VI-B α-amylase from porcine pancreas, 4-nitrophenyl α-D-glucopyranoside, acarbose, α-glucosidase from *Saccharomyces cerevisiae*, dipeptidyl peptidase IV (DPP-IV) Inhibitor Screening Kit MAK203, sitagliptin, type II lipase from porcine pancreas, sodium deoxycholate, orlistat, 4-nitrophenyl palmitate, RPMI-1640 Medium, fetal bovine serum (FBS), L-glutamine solution, sodium pyruvate, non-essential amino acids (NEAA), gentamicin, 3-[4,5-dimethylthiazol-2-yl]-2,3-diphenyl tetrazolium bromide (MTT), sodium pyruvate, β-nicotinamide adenine dinucleotide (NADH), and 2′-7′dichlorofluorescin diacetate (DCFA-DA) were purchased from Sigma-Aldrich (St. Louis, MO, USA). All other reagents were of analytical grade.

### 2.2. Obtention of Seed Flour and Analysis of Its Proximate Composition

Once cleaned, peeled, and cut into small pieces, seeds were dried at 40 °C until reaching a constant weight, ground in a domestic mill, and filtered using mesh number 60 to obtain a homogenous flour sample. The AOAC methods for moisture (934.01), fat (930.09), ash (930.05), crude fiber (934.10), and protein (978.04) were performed to determine the proximate composition of the flour. The protein value was calculated as nitrogen × 6.25, and nitrogen-free extract was estimated as the difference.

### 2.3. Protein Fractionation of Seed Flour

The separation of different protein fractions (albumin, globulin, prolamin, and glutelin) from *E. edulis* seed flour was carried out following the method reported by Chavan et al. [16], with some modifications. Firstly, the seed flour was suspended in Milli-Q water at a ratio of 1:6 (*w*/*v*), shaken for 60 min at 80 rpm, and centrifuged at 14,000× *g* for 20 min at 4 °C. The supernatant was collected and the retentate was subjected to three washing steps with Milli-Q water at a ratio of 1:3 (*p*/*v*) under the same conditions, collecting the corresponding supernatants. The pH of the supernatants was adjusted to 4.5 with 1 M HCl, and the solution was centrifuged at 14,000× *g* for 20 min at 4 °C. The retentate was collected, re-suspended in distilled water, and its pH was adjusted to 7.0 with 1 M NaOH. The albumin fraction was freeze-dried and kept at −20 °C until its further use. The extraction of globulin, prolamin, and glutelin was carried out following the same steps, only with a change in the extraction solvent: 5% NaCl for globulin, 70% ethanol for prolamin, and 0.01 M NaOH for glutelin. 

The amino acids content of protein fractions was analyzed by cation exchange chromatography using a Biochrom 30 series Amino Acid Analyser (Biochrom, Cambridge, MA, USA) after automatic pre-column derivatization of samples with OPA and measurement of the absorbance at 440 nm. The samples were previously hydrolyzed with 6 M HCl for 21 h at 110 °C. Two replicates for each sample were performed. The results were expressed as mean (g of amino acid/100 g protein).

The protein profile of protein fractions was analyzed by SDS-PAGE using 12% acrylamide gels (Merck Millipore Corp.). Samples were mixed with a sample buffer [60 mM Tris-HCl pH 6.8, 25% glycerol (*v*/*v*), 2% sodium dodecyl sulfate (SDS) (*p*/*v*), 14.4 mM 2-mercaptoethanol, and 0.1% 2-bromophenol (*p*/*v*)], heated for 4 min at 100 °C, and cooled to room temperature. 10 µg of protein was loaded onto gels and run in a Mini-Protean Tetra Cell Electrophoresis System (Bio-Rad, Richmond, CA, USA). The conditions were set at 200 V, and the gels were run for 45 min. After electrophoresis, the gels were stained with Coomassie Blue for 60 min, destained with a 10% acetic acid −10% methanol solution for 12 h and photographed using a digital camera. A pajuro protein concentrate previously obtained [15] was used as a standard. 

### 2.4. Enzymatic Hydrolysis of Pajuro Protein Fractions

A 4% (*w*/*v*) suspension of protein fractions in water was shaken at 100 rpm for 30 min and centrifuged at 2500× *g* for 10 min. The collected supernatant was heated at 90 °C for 10 min, and its pH was adjusted at 1.9 with 3 M HCl. After adding pepsin at an enzyme: substrate (E/S) ratio of 1:50 (*v*/*v*), the reaction was performed for 30 min at 37 °C with constant stirring at 80 rpm. The enzyme was inactivated by increasing the pH to 7.8 with 1 M NaOH. Then, pancreatin was added (E/S = 1:50, *v*/*v*) and the mixture was incubated for 60 min at 37 °C with constant stirring at 80 rpm. After stopping the reaction by addition of 150 mM Na_2_CO_3_ to adjust the pH to 8.5, Alcalase was added (E/S = 1/200, *v*/*v*), and the mixture was incubated for 120 min at 50 °C and 80 rpm. Aliquots were withdrawn at 0, 15, 30, 60, and 120 min, inactivating the enzyme by heating at 100 °C for 10 min. The hydrolyzates were rapidly cooled and centrifuged at 10,000× *g* for 10 min at 4 °C. The supernatants were freeze-dried and stored at −20 °C. The degree of hydrolysis (DH) was determined following the method reported by Nielsen et al. [17] The protein content was determined by the bicinchoninic acid (BCA, Pierce, Rockford, IL, USA) method, using BSA as a standard protein.

### 2.5. Antioxidant Activity

The ABTS^•+^ scavenging activity was determined according to the enhanced improved ABTS^•+^ protocol described by Re et al. [18]. A volume of 980 μL of diluted ABTS^•+^ solution and 20 μL of either PBS (blank), Trolox (2–25 µM) (standard) or sample were mixed, and the absorbance was measured at 734 nm after 7 min-incubation at room temperature. To calculate the Trolox equivalent antioxidant capacity (TEAC), the gradient of the plot of the percentage inhibition of absorbance versus protein concentration was divided by the gradient of the plot for Trolox. TEAC values were expressed as µmol Trolox equivalent (TE)/mg of protein. 

The oxygen radical absorbance capacity (ORAC) was determined following the protocol described by Hernández-Ledesma et al. [19], with some modifications. Briefly, the mixture (200 µL) containing FL (30 nM), AAPH (12 mM), and either antioxidant [Trolox (0–5 nM) or sample (at different concentrations)], was incubated at 37 °C in 75 mM phosphate buffer (pH 7.4). Then, we recorded the fluorescence at 485 and 520 nm of excitation and emission, respectively, at every 2 min for 120 min in an Infinite M200 Pro plate reader (Tecan Group AG, Männendorf, Switzerland) controlled by Icontrol software version 1.11.10. ORAC values were expressed as µmol TE/mg of protein.

### 2.6. Angiotensin-Converting Enzyme (ACE) Inhibitory Activity

The in vitro ACE inhibitory activity of hydrolyzates was measured using the methodology described by Hayakari and coworkers [20], with some modifications. The reaction mixture (200 µL) containing 1 mU ACE, 1.25 mM HHL, and sample or captopril (used as standard) at different concentrations was incubated at 37 °C for 60 min. The reaction was stopped by heating at 100 °C for 10 min. A volume of 100 µL of a 3% solution of trichloro-5-triazine/dioxan was added, and the mixture was shaken and centrifuged at 1000× *g* for 10 min. Then, the absorbance was measured at 382 nm in an Infinite M200 Pro plate reader (Tecan Group AG). The activity was expressed as % (compared with control) and IC_50_ or protein concentration required to inhibit the ACE activity by 50%.

### 2.7. In Vitro Anti-Diabetic Activity

The α-amylase inhibition assay was carried out following the methods previously described [21,22], with some modifications. The reaction mixture (200 µL) containing α-amylase (800 mU), 0.125% starch, and sample or standard (at different concentrations) in 0.1 M phosphate buffer (pH 6.9) was incubated at 37 °C for 30 min. A volume of 100 µL of a solution containing 1% 3,5-dinitrosalicylic acid and 30% sodium potassium tartrate in 0.4 M NaOH was added, shaking the mixture, and incubating it at 100 °C for 10 min. The absorbance was measured at 540 nm in an Infinite M200 Pro plate reader (Tecan Group AG). The inhibition percentage was calculated relative to the negative control having 100% enzyme activity.

The α-glucosidase inhibition assay was performed following previous methods [23,24], with some modifications. Briefly, 200 µL of reaction mixture containing 10 mU α-glucosidase, 5 mM 4-nitrophenyl-α-D-glucopyranoside, and sample or standard acarbose (at different concentrations) in 0.1 M phosphate buffer (pH 6.9) was incubated at 25 °C for 30 min, reading the absorbance each 2 min at 405 nm in the Infinite M200 Pro plate reader (Tecan Group AG). Percent inhibition was calculated relative to the negative control having 100% enzyme activity.

The DPP-IV inhibitory activity was determined using the DPP-IV Inhibitor Screening Kit MAK203. The reaction mixture (100 µL), containing 5 µL of enzymatic solution, sample or standard sitagliptin (at different concentrations), and 5 µL of substrate, was incubated at 37 °C for 30 min, and the fluorescence was recorded each 2 min at λexcitation and λemission of 360 and 460 nm, respectively, in the Infinite M200 Pro plate reader (Tecan Group AG). The results were expressed as IC_50_ value or protein concentration needed to inhibit 50% DPP-IV activity.

### 2.8. Pancreatic Lipase Inhibitory Activity

The pancreatic lipase inhibitory assay was performed following methods previously described [25,26], with some modifications. The reaction mixture (200 µL) contained 50 mM sodium deoxycholate, 0.2 mM CaCl_2_, 12 mU type 2 pancreatic lipase, sample or standard orlistat (at different concentrations), and 0.125 mM substrate 4-nitrophenyl palmitate in 50 mM phosphate buffer (pH 8.0). The mixture was incubated at 37 °C for 60 min, and the absorbance at 405 nm was measured every 2 min in the Infinite M200 Pro plate reader (Tecan Group AG). The inhibition percentage was calculated relative to the negative control having 100% enzyme activity.

### 2.9. Protective Effects in SH-SY5Y Cells

#### 2.9.1. Cell Culture

Human neuroblastoma SH-SY5Y cells were obtained from the American Type Culture Collection (ATCC, HTB-38, Rockville, MD, USA). The cells were maintained in RPMI-1640 medium supplemented with 10% FBS, 2 mM L-glutamine solution, 1 mM sodium pyruvate, 1 mM non-essential amino acids, and 50 ug/mL gentamicin. Cells were incubated at 37 °C under a 5% CO_2_/95% air at constant humidity.

#### 2.9.2. Effects on Cell Viability

Cell viability was determined using the MTT assay. SH-SY5Y cells were seeded onto 96-well plates at a density of 2 × 10^4^ cells/well in complete medium with 1% FBS and incubated for 16 h at 37 °C. Afterwards, culture medium was removed, and the sample was added (at concentration between 0.25 and 2.0 mg/mL), incubating the plate for 24 h. After removing the supernatant, a MTT solution (2 mg/mL in PBS) was added, and the plate was incubated for 60 min at 37 °C. The supernatant was aspirated, insoluble formazan crystals formed were dissolved in dimetilsulfoxide (DMSO), and the absorbance was measured at 570 nm in the Infinite M200 Pro plate reader (Tecan Group AG). The results were expressed as percentage of the control, considered as 100%.

#### 2.9.3. Protective Effects against Oxidative Stress Induced by FeSO_4_

For this assay, SH-SY5Y cells were seeded onto 48-well plates at a density of 4 × 10^4^ cells/well in complete medium with 1% FBS and incubated for 16 h at 37 °C. The medium was aspirated, and cells were washed with PBS. Then, hydrolyzate (concentrations of 0.1, 0.5, and 1.0 mg/mL) and 10 mM glucose were added, and cells were incubated for 30 min. Once the supernatant was removed, the cells were maintained in complete medium with 1% FBS for 6 h at 37 °C. Intracellular ROS levels were quantified following the method described by LeBel et al., using dichlorofluorescin (DCFH) as a fluorescent probe [27]. The medium was aspirated, and 100 µL of a solution containing 10 mM DCFA-DA and 10 mM glucose in PBS was added to the wells, and the plate was incubated at 37 °C for 30 min. Then, the supernatant was discarded, cells were washed with PBS, and 200 µM FeSO_4_ in complete medium with 1% FBS was added, measuring the fluorescence after 60 min-exposure to the ROS inductor in the Infinite M200 Pro plate reader (Tecan Group AG). The excitation and emission wavelengths were 485 and 530 nm, respectively. The results were expressed as ROS levels (% compared with the control, considered as 100%).

#### 2.9.4. Determination of Thiobarbituric Acid Reaction Substances (TBARS)

SH-SY5Y cells were seeded onto 60 mm dishes at a density of 7.5 × 10^5^ cells/dish in complete medium with 10% FBS and incubated for 72 h. Then, the supernatant was discarded and the hydrolyzate, dissolved in PBS with 10 mM glucose, was added to the wells (final concentration of 0.5, 1.0, and 1.5 mg/mL). The plate was incubated for 30 min and then the cells were washed with PBS and stimulated with 200 µM FeSO_4_ for 3 h in complete medium with 1% FBS. Afterwards, both the supernatant and cell pellet were collected. The cell pellet was resuspended in 50 mM phosphate buffer (pH 7.4) and subjected to ultrasonication. 250 μL of 1% phosphoric acid and 75 μL of TBA were added to 30 μL of cellular suspension, and the mixture was incubated 100 °C for 45 min. Once cooled and centrifuged at 3000× *g* for 5 min a 4 °C, the fluorescence was measured at excitation and emission wavelengths of 485 and 530 nm, respectively, in the Infinite M200 Pro plate reader (Tecan Group AG). A standard curve with malondialdehyde (MDA) was used. The results were expressed as nmol MDA/mg protein.

#### 2.9.5. Determination of Lactate Dehydrogenase (LDH) Activity

A volume of 100 µL of supernatant previously collected was mixed with 100 µL of the reaction solution containing 0.18 mM sodium pyruvate and 0.6 mM NADH in phosphate buffer (50 mM, pH 7.4). After 2 min-incubation, the fluorescence was measured at excitation and emission wavelengths of 360 and 460 nm, respectively, in the Infinite M200 Pro plate reader (Tecan Group AG). The results were expressed as LDH activity (% compared with the control, considered as 100%).

### 2.10. Statistical Analysis

All data were analyzed in 3 independent experiments, and results were expressed as the mean ± standard deviation (SD) or standard error of the mean (SEM). Data were analyzed using a one-way analysis of variance (ANOVA) followed by the Tukey test for multiple comparisons. All analyses were run with the program GraphPad Prism v.6. or SigmaPlot 14.5 Statistical, significance was defined as *p* < 0.05.

## 3. Results and Discussion

### 3.1. Obtention and Fractionation of E. edulis Seed Flour

The values of ash, fat, and fiber content determined in the *E. edulis* seed flour were 39.5 g/kg, 13.1 g/kg, and 58.9 g/kg, respectively. The protein content of the flour (186.0 g/kg) was comparable to that (184 g/kg) determined by Arango Bedoya et al. [12]. The flour was fractionated by using different solvents into albumin, globulin, prolamin, and glutelin fractions, obtaining yield values of 3.93, 0.25, 0.02, and 3.80%, respectively. Table 1 shows the amino acid composition of the flour and extracted fractions.

The amino acid Trp was not identified under the conditions used because of its destruction by acid hydrolysis. In the flour, Leu, Val, and Lys were the most abundant essential amino acids (EAA), with values of 2.68 ± 0.24, 2.11 ± 0.13, and 2.06 ± 0.18 g/100 g of protein, respectively. Asp + Asn and Glu + Gln were the most abundant non-essential amino acids (NEAA), with values of 4.78 ± 0.37 and 5.50 ± 0.55 g/100 g of protein, respectively, as it has been reported for other legumes such as pea [28]. The ratio of EAA to total amino acid (TAA) was 40%, and the ratio of EAA to NEAA was 66% that were similar to the protein reference pattern (EAA/TAA, 40%; EAA/NEAA, 60%) raised by FAO/WHO. An increase in the TAA content was observed for albumin (111.7%), globulin (105.9%), and glutelin (48.2%) fractions. However, a reduction by 22.7% was observed for prolamin fraction. Nevertheless, the ratios EAA/TAA and EAA/NEAA were maintained in four protein fractions.

**Figure 1:**
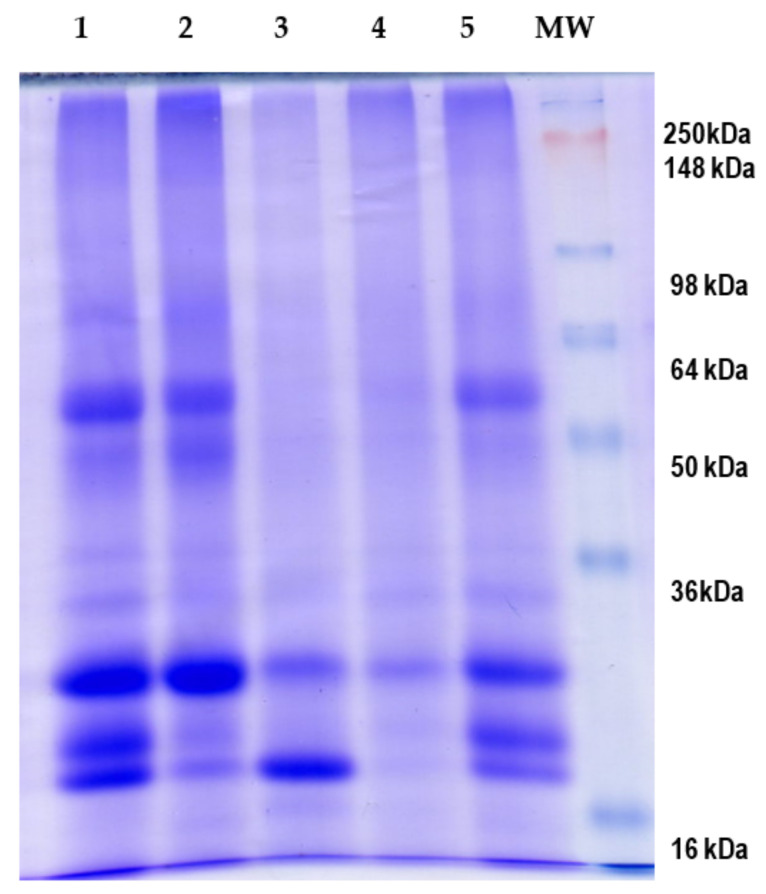
SDS-PAGE analysis of protein fractions obtained from *Erythrina edulis* (pajuro): (1) albumin; (2) globulin; (3) prolamin; (4) glutelin. (5) Protein concentrate. MW: molecular weight marker SeeBlue™ Plus2 Prestained.

SDS-PAGE was run to analyze the protein profile of the protein concentrate and fractions (Figure 1). This analysis revealed a high number of bands from 16 to 90 kDa, being bands corresponding to proteins with molecular weight of 18, 20, 25, 50, and 58 kDa clearly visible. The intensity of the 25 kDa and 18-kDa bands was more intense in the globulin and prolamin fractions, respectively.

### 3.2. Enzymatic Hydrolysis of Pajuro Protein Fractions

**Figure 2:**
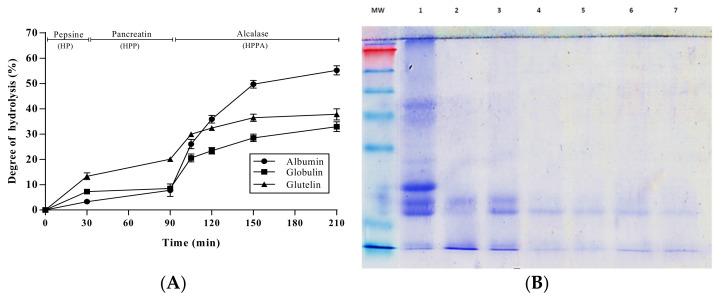
(**A**) Degree of hydrolysis (%) of the hydrolyzates from *Erythrina edulis* (pajuro) seed-derived albumin, globulin, and glutelin fractions after the action of pepsin for 30 min (PH), pepsin 30 min + pancreatin 60 min (PPH), and pepsin 30 min + pancreatin 60 min + Alcalase at different times (PPHA15, PPHA30, PPHA60, and PPH120). (**B**) SDS-PAGE analysis of (1) *Erythrina edulis* (pajuro) seed albumin fraction; (2) hydrolyzate by pepsin for 30 min (PH); (3) hydrolyzate by pepsin for 30 min and pancreatin for 60 min (PPH); (4) hydrolyzate by pepsin for 30 min, pancreatin for 60 min, and Alcalase for 15 min (PPHA15); (5) hydrolyzate by pepsin for 30 min, pancreatin for 60 min, and Alcalase for 30 min (PPHA30); (6) hydrolyzate by pepsin for 30 min, pancreatin for 60 min, and Alcalase for 60 min (PPHA60); (7) hydrolyzate by pepsin for 30 min, pancreatin for 60 min, and Alcalase for 120 min (PPHA120). MW: molecular weight marker SeeBlue™ Plus2 Prestained.

Albumin, globulin, and glutelin fractions obtained from pajuro seed flour were sequentially hydrolyzed by pepsin for 30 min, pancreatin for 60 min, and alcalase for 15, 30, 60, and 120 min. Digestion efficiency was monitored by the estimation of the DH. Figure 2A shows the DH of hydrolyzates obtained after pepsin hydrolysis (PH), pepsin + pancreatin hydrolysis (PPH), and pepsin + pancreatin + alcalase hydrolysis at different times (PPHA15, PPHA30, PPHA60, and PPHA120). As shown in the Figure 2A, the DH slightly increased during the pepsin + pancreatin hydrolysis of the albumin and globulin fractions. The DH value reached in the albumin PPH (7.81%) was lower to that determined recently by Moscoso-Mújica et al. for hydrolyzates obtained after pepsin-pancreatin co-incubation with albumin from *Chenopodium pallidicaule* Aellen (kanihua) for 1 h (DH of 22%) [29]. In the case of glutelin fraction, the increase was moderated, reaching a DH value for PPH of 20.07%. This value was similar to those reported for raw peanut protein hydrolyzed by pepsin for 30 min and pancreatin for 24 h (21.4%) [30], and for *Phaseolus lunatus* (lima bean) protein hydrolyzed by pepsin for 45 min and pancreatin for 45 min (22.09%) [31].

Moreover, the DH of glutelin PPH was higher to that described for the kanihua-derived glutelin fraction hydrolyzed for 2 h with both digestive enzymes (15.0%) [28], although it was lower than that reported by Zhan et al. for sacha-inchi-derived glutelin hydrolyzed for 6 h with pepsin + pancreatin (28.6%) [32]. The differences observed could be due to the conditions of the enzymatic reaction and the characteristics of the protein source.

The PPHs were subjected to further hydrolysis with Alcalase, observing a gradual increase of DH with incubation time, reaching values of 55.23, 32.94, and 37.82% at the end of hydrolysis of albumin, globulin, and glutelin, respectively. The observed differences could be due to the protease specificity of Alcalase and the protein source [33]. The highest increase in DH was observed in the albumin fraction, indicating the higher susceptibility of this protein to the enzymatic action. The broad specificity of Alcalase has been demonstrated, thus it is known that this microbial enzyme hydrolyzes most peptide bonds, releasing peptides with hydrophobic (Trp, Leu, Ile, Val, and Met) and aromatic (Phe and Tyr) amino acids [34]. The higher content of these amino acids in the albumin fraction (Table 1) could be responsible for its higher susceptibility to the microbial enzyme in comparison with globulin and glutelin fractions. Proteins from other plant species have also been found to be susceptible to Alcalase hydrolysis. Thus, protein from *Vigna unguiculata* was partially hydrolyzed by this microbial enzyme, reaching a DH value of 53.0% after 90 min of incubation [35]. Similarly, globulins and albumins from eight chickpea (*Cicer arietinum* L.) genotypes were found to be susceptible to Alcalase, and hydrolyzates obtained after 90 min showed DH values between 33.8 and 42.6% [36]. The susceptibility of albumin to enzymatic hydrolysis was confirmed using a SDS-PAGE analysis. As shown in Figure 2B, the intensity of all bands visible in the non-hydrolyzed sample gradually decreased over the course of hydrolysis. Even bands of 58 and 25 kDa disappeared after the action of pepsin for 30 min. Only the band of 20 kDa remained partially intact at the end of the hydrolytic process, indicating the partial resistance of this protein to the action of both digestive and microbial enzymes. 

### 3.3. Antioxidant Activity of Pajuro Protein Fractions

**Figure 3:**
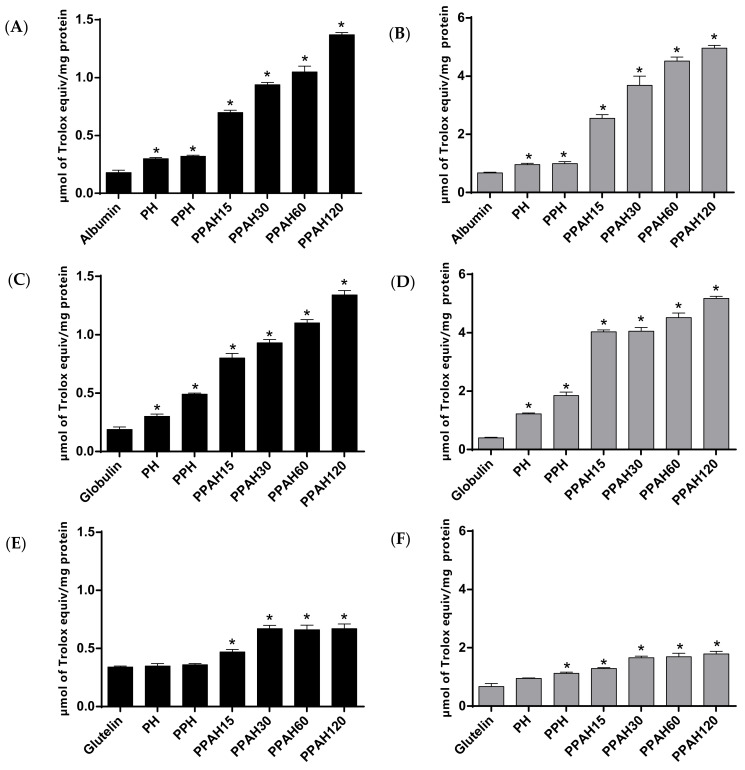
ABTS^•+^ (**A**,**C**,**E**) and peroxyl (**B**,**D**,**F**) radical scavenging capacity (expressed as µmol Trolox equivalents/mg of protein) of hydrolyzates from (**A**,**B**) albumin, (**C**,**D**) globulin, and (**E**,**F**) glutelin with pepsin (PH), pepsin + pancreatin (PPH), and pepsin + pancreatin + alcalase for 15 min (PPHA15), 30 min (PPHA30), 60 min (PPHA60) and 120 min (PPHA120). * *p* > 0.05 vs. protein fraction.

Currently, the use of more than one method to evaluate the antioxidant activity of a compound is recommended. Thus, two biochemical assays (ABTS and ORAC) were selected to measure the ABTS^•+^ and peroxyl radical scavenging capacity, respectively, of different hydrolyzates obtained from pajuro protein fractions (Figure 3A,B). Although intact protein fractions showed a slight radical scavenging capacity, their sequential proteolysis considerably increased it. The ABTS assay quantifies the suppressive capacity of an antioxidant against the ABTS^•+^ radical. When added to medium containing this radical, the peptides released during hydrolysis act as electron donors, converting this radical cation into the non-radical ABTS. As shown in Figure 3A,C,E, proteolysis of pajuro protein fractions increased ABTS^•+^ radical scavenging activity between 2- and 7-times, compared with non-hydrolyzed fractions. Albumin and globulin hydrolyzates obtained at the end of complete hydrolytic process showed the highest TEAC values (1.37 µmol TE/mg of albumin and 1.34 µmol TE/mg of globulin), without differences between both fractions. These values were higher than those previously determined in the hydrolyzate of pajuro protein concentrate with Alcalase [13]. The higher ABTS^•+^ radical scavenging capacity of the hydrolyzates obtained after the combined action of three enzymes (pepsin, pancreatin, and Alcalase) in comparison with those obtained only by pepsin could be due to the higher efficiency of the hydrolytic process. Thus, Chirinos et al. reported the higher antioxidant activity of Alcalase-Flavourzyme hydrolyzates from tarwi (*Lupinus mutabilis*) than that of Alcalase hydrolyzates [37]. Similarly, other works have found higher ABTS^•+^ radical scavenging capacity of soybean [38], cañihua (*Chenopodium pallidicaule*) [39], and kiwicha (*Amaranthus caudatus*) [40] protein hydrolyzates using Alcalase combinations with other enzymes compared with only Alcalase.

ORAC assay measures the ability of an antioxidant compound to break the chain reactions of thermally peroxyl radicals produced from AAPH. It is considered as one of the best methods for routine in vitro antioxidant capacity analysis of dietary supplements and food compounds, which combines peroxide radicals with physiological conditions, and has a wide application potential [41]. Pepsin hydrolyzates of pajuro protein fractions showed moderate ORAC values between 0.95 and 1.22 µmol TE/mg of protein (Figure 3B,D,F).

After the sequential hydrolysis by pepsin and pancreatin, the antioxidant activity slightly increased in the albumin and glutelin hydrolyzates while the increase was more notable in the globulin hydrolyzate. Newly, the combined action of digestive and microbial enzymes resulted in hydrolyzates with potent peroxyl radicals scavenging capacity, obtaining ORAC values of 1.79, 4.96, and 5.18 µmol TE/mg of protein for glutelin, albumin, and globulin fractions, respectively. The highest values corresponded to albumin and globulin-derived hydrolyzates content in antioxidant amino acids (Tyr, Phe, Pro, Ala, His, Asp, Leu and Met), which are capable of acting as effective proton/hydrogen donors [42] representing 47% of TAA, suggesting the potential contribution of these residues on the antioxidant activity shown by the hydrolyzates. Albumin hydrolyzates from mung bean (*Vigna radiata*) have also been recently reported as a potent source of antioxidant peptides [43].

### 3.4. In Vitro Anti-Hypertensive and Anti-Diabetic Activity of Pajuro Albumin Hydrolyzates

Because of the high antioxidant activity demonstrated by albumin-derived hydrolyzates, they were selected to evaluate their multifunctionality. Firstly, the anti-hypertensive and anti-diabetic effects were evaluated in vitro through inhibition of ACE, α-amylase, α-glucosidase, DPP-IV, and pancreatic lipase activities (Table 2).

ACE is an enzyme that catalyzes the degradation of angiotensin I to produce angiotensin II with potent vasoconstrictor activity, and the cleavage of vasodilator bradykinin, thus promoting the increase of the blood pressure [44]. Thus, ACE inhibitory peptides are considered as a useful strategy for preventing hypertension. As observed in Table 2, the intact albumin fraction did not show any inhibitory effect on ACE. However, after its sequential hydrolysis by digestive and microbial enzymes, the inhibitory capacity gradually increased, reaching 83.60% inhibition when albumin-PPHA120 was analyzed at 100 µg protein/mL. The IC_50_ calculated for this hydrolyzate was 50.65 µg protein/mL. Although this value was higher than that measured for Captopril, used as standard in this assay (6.81 µg/mL), it was much lower than those reported for hydrolyzates from other plant proteins. As examples, Alcalase-hydrolyzates of *Lupinus angustifolius* protein showed IC_50_ values ranged from 100–210 µg protein/mL [45], and the tarwi protein hydrolyzate by an Alcalase-Neutrase combination for 180 min showed an IC_50_ value of 110 µg protein/mL [37].

The antidiabetic activity of albumin-derived hydrolyzates was determined by evaluating the inhibition of metabolic enzymes α-amylase, α-glucosidase, and DPP-IV. α-amylase and α-glucosidase are the primary enzymes implied in the digestion of dietary starch, releasing oligosaccharides that can be further broken down into glucose, which is rapidly absorbed by the body. Therefore, blocking these enzymes results in a reduced absorption rate of sugars and helps to manage both body weight and blood sugars [46]. Undigested albumin at concentration of 150 µg protein/mL inhibited 22.80% α-amylase, without showing any effects on the rest of enzymes. Acarbose, used as standard in the α-amylase and α-glucosidase inhibition assays, showed 87.44% and 70.21% inhibition, respectively, at concentrations of 10 mM. Once completely hydrolyzed, the final digest showed slight inhibitory effects on α-amylase and α-glucosidase that could be due to the low protein concentration assayed (150 µg protein/mL). On the contrary, other studies have reported higher inhibition rates at higher concentrations of protein. Thus, incubation of 225 µg protein/mL of yellow field pea (*Pisum sativum* L.) protein hydrolyzates with α-amylase resulted in ≈31% inhibition of the enzyme while a concentration of 20 mg/mL was needed to inhibit 53.4% of α-glucosidase activity [47]. Mojica and de Mejia reported 53.4% α-amylase inhibition for Alcalase hydrolyzate of black bean proteins at a final concentration of 330 mg hydrolyzate/mL [48]. In the case of α-glucosidase inhibitory activity, gastrointestinal digests from quinoa protein showed and IC_50_ value of 1.81 mg protein/mL [49], and Alcalase hemp seed proteins hydrolyzates reached 50% enzyme inhibition at concentrations higher than 5 mg/mL [50]. A recent study reported inhibitory values of 38.9% (α-amylase) and 48.8% (α-glucosidase) for eggplant leaf protein hydrolyzates at 500 µg hydrolyzate/mL [51].

In contrast to the moderate effects observed on α-amylase and α-glucosidase, the pajuro albumin hydrolyzates showed potent inhibitory effects on DPP-IV. This enzyme is responsible for degradation and inactivation of gut incretin hormones that act after food ingestion by stimulating glucose-dependent insulin secretion in the pancreatic β-cells [52,53]. Specific DPP-IV inhibition has been demonstrated to increase the half-life of circulating incretins, to reduce plasma glucose levels, and to improve glucose tolerance [54]. Undigested albumin (300 µg protein/mL) did not show any effects on DPP-IV. This result agrees previous studies that had reported null or weak inhibitory effects for intact proteins from plant and animal sources. Thus, intact walnut protein at 500 µg/mL showed DPP-IV inhibition rates lower than 5% [55]. In another recent study, low DPP-IV inhibitory activity was also reported for undigested amaranth protein isolate as reflected by an IC_50_ value of 8.22 mg/mL [56]. Similarly, a previous study had reported low DPP-IV inhibitory effects for intact amaranth glutelins [57]. In this last study, inhibitory activity increased in a dose-dependent manner when glutelins were subjected to trypsin hydrolysis. In our study, the inhibitory effects on DPP-IV were hydrolysis-dependent, reaching 35.73% inhibition after the action of digestive enzymes and 64.81% at the end of sequential hydrolysis (Table 2). The IC_50_ value calculated by albumin-PPHA120 was 230.57 µg protein/mL. This value was similar to that reported for gastrointestinal digests from quinoa protein (250 µg protein/mL) [49] and higher than that reported by for walnut protein hydrolyzates by Alcalase (900 µg/mL) [55]. The differences found among studies could be due to the protein source and the type of enzymes involved in the hydrolysis [58,59].

Since pancreatic lipase is a crucial enzyme involved in the intestinal digestion of dietary triacylglycerols, a major source of excess calorie intake, its inhibition results in reduction of fat digestion and absorption, and consequently in moderate long-term reductions in body weight [60]. Therefore, lipase inhibitors are considered as useful in the management of obesity [61]. In our study, no inhibitory effects were observed for both undigested and digested pajuro albumins that could be due to the low concentration assayed (150 µg protein/mL). Previous studies have reported inhibitory effects on this enzyme at much higher doses. Thus, the IC_50_ value reported for Alcalase hydrolyzates from pea protein was 3.98 mg/mL [47], and values determined in black bean proteins hydrolyzates with pepsin/pancreatin or alcalase were 3.21 and 1.23 mg/mL, respectively [62].

### 3.5. Neuroprotective Effects of Pajuro Albumin Hydrolyzates

**Figure 4:**
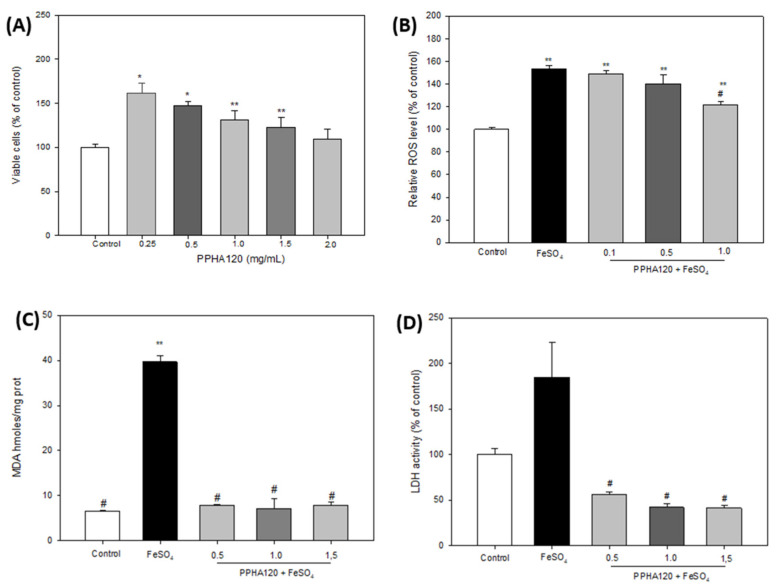
(**A**) Viable SH-SY5Y cells (% of control) after their treatment with the pajuro albumin hydrolyzate PPHA120 at different concentrations (0.25–2 mg/mL). (**B**) ROS levels (% of control) after the treatment of SH-SY5Y cells with FeSO_4_ in the absence and presence of PPHA120 at different concentrations (0.1–1.0 mg/mL). (**C**) MDA levels (nmoles/mg protein) after the treatment of SH-SY5Y cells with FeSO_4_ in the absence and presence of PPHA120 at different concentrations (0.5–1.5 mg/mL). (**D**) LDH activity (% of control) after the treatment of SH-SY5Y cells with FeSO_4_ in the absence and presence of PPHA120 at different concentrations (0.5–1.5 mg/mL). * *p* < 0.05 or ** *p* < 0.001 vs. control (non-treated cells), # *p* < 0.001 vs. FeSO_4_-treated cells.

Because of the limitations associated with the use of synthetic compounds against neurodegenerative disorders, the interest has focused on naturally derived compounds with potential neuroprotective properties [63]. Thus, we aimed to evaluate the neuroprotective effects of pajuro albumin hydrolyzates against FeSO_4_-induced neurotoxicity in the SH-SY5Y cell line. In our study, these hydrolyzates have been demonstrated to scavenge radicals in both ABTS and ORAC assays (Figure 3). The excessive production of free radicals in the brain together the accumulation of transition metals has been reported to contribute on the neuronal cell death [64]. Therefore, scavenging free radicals could inhibit neurodegenerative diseases [65]. Firstly, MTT analysis was performed to evaluate the cytotoxicity of the albumin hydrolyzates against neuroblastoma SH-SY5Y cells under basal conditions at doses ranged from 0.25 to 2 mg/mL. As shown in Figure 4A, the highest dose (2 mg/mL) of the albumin-PPHA120 did not affect the cell viability. However, lower doses resulted in a significant increase of the viability reaching 161.41% viable cells (compared with non-treated cells) when cells were treated with 0.25 mg/mL of the hydrolyzate.

Oxidative damage induces increase of intracellular ROS, which leads to cell death [66]. Therefore, we investigated the protective effects of the albumin hydrolyzate against the oxidative damage induced by FeSO_4_ through quantification of the intracellular ROS levels using fluorescence with DCFA-DH (Figure 4B). In this assay, concentrations of 0.25, 0.5, and 1 mg/mL of albumin-PPHA120 were used. FeSO_4_ stimulation resulted in a significant increase of ROS levels up to 153.31% (compared to non-induced cells). However, treatment of induced cells with the hydrolyzate reverted the oxidative damage in a dose-dependent manner although the ROS levels quantified at the highest concentration assayed were still above control (121.58%). Although the data are still limited, some food-derived hydrolyzates have also been found to decrease ROS levels in injured nerve cells. Thus, Sheng et al. reported protective effects of defatted walnut (*Juglans regia* L.) meal hydrolyzate against oxidative damage induced by H_2_O_2_ in SH-SY5Y cells [67]. A peptide (SGGY) identified in the walnut protein after simulating its gastrointestinal digestion was also demonstrated to exert effective protection in this cell model [68]. Similarly, Lee & Hur demonstrated that peptide fractions derived from beef protein hydrolyzates with alkaline-AK and papain decreased ROS levels compared to H_2_O_2_-treated SH-SY5Y cells [69]. Next, these authors extracted the <3 kDa fraction from the alkaline-AK hydrolyzate and evaluated their protective effects on induced neuronal cells through other mechanisms of action [70]. They demonstrated the neuroprotective effects of the fraction by increasing cell viability, inhibiting nitric oxide production and fragmentation of cell nuclei, increasing the mitochondrial membrane potential, and reducing apoptosis. In addition to the effects on ROS generation, we aimed at evaluating the effects of the albumin hydrolyzate through other mechanisms. Thus, their effects on lipidic peroxidation and LDH levels were investigated. As shown in Figure 4C, cell induction with FeSO_4_ resulted in a significant increase of MDA levels that was reverted by the albumin-PPHA120 at all assayed concentrations. LDH is considered an important index to detect cell death because it is released into the culture medium when the cell membrane is damaged [71]. In our study, we found that the level of LDH was significantly increased in FeSO_4_-induced cells (184.65% compared to non-induced cells), while treatment with the hydrolyzate the LDH levels in the supernatant were all significantly reduced in SH-SY5Y cells, reaching values lower than that of control cells (42.16% at dose of 1.0 mg/mL) (Figure 4D).

## 4. Conclusions

In the present study, albumin, globulin, glutelin, and prolamin fractions were separated from *E. edulis* flour and hydrolyzed by a sequential process with digestive and microbial enzymes. Among hydrolyzates produced by the combined action of pepsin, pancreatin, and Alcalase on albumin fraction showed potent radical scavenging and ACE inhibitory activity, and anti-diabetic properties through inhibition of α-amylase, α-glucosidase, and DPP-IV enzymes. It also exerted a notable neuroprotective effect in a neuroblastoma cell model induced by FeSO_4_ through modulation of oxidative stress-associated biomarkers. These findings point pajuro albumin out as a promising source of multifunctional peptides with high value in the formulation of functional foods and/or nutraceuticals for health promotion and prevention of chronic disorders associated to oxidative stress, hypertension, and metabolic alterations. Further research would be needed to isolate and identify the peptides responsible for the observed effects and confirm their bioavailability and bioactivity. 

## Figures and Tables

**Table 1:** Amino acid composition (g/100 g of protein) of Erythrina edulis flour and its protein fractions.

Amino Acid	Content (g/100 g Protein)					
	Seed Flour	Albumin	Globulin	Glutelin	Prolamin	FAO
Essential						
Lys	2.06	4.39	4.48	3.20	1.88	5.2
Trp	n.d.	n.d.	n.d.	n.d.	n.d.	0.7
Phe	1.98	4.25	4.01	3.12	1.57	4.6 ^a^
Tyr	1.23	3.82	3.92	2.63	0.74	
Met	0.38	1.18	1.22	0.93	0.39	2.6 ^b^
Cys	0.89	1.02	1.03	0.49	0.45	
Thr	1.19	2.26	2.41	1.82	0.89	2.7
Leu	2.68	6.11	5.98	4.47	2.43	6.3
Ile	1.16	2.56	2.50	1.77	1.08	3.1
Val	2.11	3.62	3.38	2.36	1.68	4.2
Non essential						
Asx ^c^	4.78	9.57	8.99	6.50	3.44	
Glx ^d^	5.50	11.98	11.65	8.05	4.15	
Ser	2.24	5.11	5.16	3.57	1.66	
His	1.50	1.90	1.88	1.59	0.68	
Arg	1.05	3.58	3.63	2.45	0.86	
Ala	1.68	3.30	2.99	2.62	1.59	
Pro	2.19	4.17	3.98	2.74	1.58	
Gly	1.69	3.83	3.45	2.55	1.43	
TAA	34.31	72.65	70.66	50.86	26.50	
HAA	14.30	30.03	29.01	21.13	11.51	
AAA	3.21	8.07	7.93	5.75	2.31	

n.d. not determined; ^a^ Phe + Tyr; ^b^ Met + Cys; ^c^ Asp + Asn; ^d^ Glu + Gln. HAA: hydrophobic amino acids (Ala, Val, Ile, Leu, Tyr, Phe, Trp, Met, Pro, and Cys); TAA: total amino acids; AAA: aromatic amino acids (Phe, Trp, and Tyr). Data are the mean of two determinations.

**Table 2:** In vitro anti-hypertensive and anti-diabetic activities of pajuro albumin-derived hydrolyzates obtained by hydrolysis with pepsin (PH), hydrolysis with pepsin and pancreatin (PPH), and hydrolysis with pepsin, pancreatin, and Alcalase for 15 min (PPHA15), 30 min (PPHA30), 60 min (PPHA60) and 120 min (PPHA120).

Sample	ACE Inhibition (%) ^a^	α-Amylase Inhibition (%) ^b^	α-Glucosidase Inhibition (%) ^b^	DPP-IV Inhibition (%) ^c^	Pancreatic Lipase Inhibition (%) ^a^
Albumin	0.10 ± 0.04	22.80 ± 6.85	1.61 ± 0.18	2.31 ± 1.48	n.d.
Albumin PH	13.45 ± 1.88 *	n.d.	n.d.	16.16 ± 1.48 *	n.d.
Albumin PPH	20.85 ± 1.06 *	n.d.	n.d.	35.73 ± 1.35 *	n.d.
Albumin PPHA15	76.75 ± 4.48 **	n.d.	n.d.	60.69 ± 6.70 **	n.d.
Albumin PPHA30	64.20 ± 6.15 **	n.d.	n.d.	58.19 ± 3.13 **	n.d.
Albumin PPHA60	82.22 ± 5.51 **	n.d.	n.d.	58.61 ± 9.51 **	n.d.
Albumin PPHA120	83.60 ± 0.09 **	14.60 ± 1.32	6.06 ± 1.61	64.81 ± 5.31 **	n.d.
Positive control	6.81 ± 0.04	87.44 ± 1.61	70.21 ± 0.47	0.86 ± 0.01	65.92 ± 2.83

^a^: the percentage of ACE inhibition was determined at 100 µg protein/mL; ^b^: the percentage of α-amylase, α-glucosidase, and pancreatic lipase inhibition was determined at protein concentration of 150 µg protein/mL; ^c^: the percentage of DPP-IV inhibition was determined at protein concentration of 300 µg protein/mL. Positive Controls: Captopril for ACE inhibition (IC_50_); acarbose for α-amylase and α-glucosidase activity (% inhibition at 10 mM); sitagliptin for DPP-IV inhibition (IC_50_); orlistat for pancreatic lipase inhibition (% inhibition at 0.5 µM). * *p* < 0.05 or ** *p* < 0.001 vs. albumin. The values corresponded to the mean of three replicates.

## Data Availability

Data is contained within the article.
